# Multidimensional bandwagon effect and dual-process decision making: An integrated model of the theory of planned behavior from the perspective of American millennials’ travel intention

**DOI:** 10.1016/j.heliyon.2024.e41375

**Published:** 2024-12-21

**Authors:** Roannayutt Oan-Oon, Therdchai Choibamroong

**Affiliations:** Graduate School of Tourism Management, National Institute of Administration Development, Bangkok, Thailand

**Keywords:** Bandwagon effect, Destination choice, Millennials, Dual-process decision-making, Theory of planned behavior, Behavior economics, Heuristics, Cognitive biases

## Abstract

This study addresses the imperative need for an updated approach that incorporates evolving psychological insights and economic theories to better understand decision-making processes in the tourism sector. By integrating the bandwagon effect with the theory of planned behavior (TPB), the study aims to gain deeper insights into the intention-forming processes of American millennials during the pre-trip stage when considering a visit to Thailand. The research amalgamates principles from behavioral economics and traditional psychological theory within the dual-process framework, providing a comprehensive understanding of how American millennials determine their visit intention. Through empirical analysis employing Partial Least Squares Structural Equation Modeling (PLS-SEM) in a post-pandemic context, the study reveals that the multidimensional bandwagon effect significantly and synergistically influences American millennials' travel intention, attitude, and subjective norm, supporting a dual-process decision-making model. These findings elucidate how American millennials navigate heuristics and cognitive biases based on the theory of planned behavior framework, offering valuable insights for destination management organizations to refine their marketing and communication strategies effectively.

## 1 Introduction

The remarkable success of Thailand's meticulously planned and executed tourism industry, which attracted nearly 40 million visitors and contributed approximately 11 % to the nation's GDP in 2019, has recently encountered significant challenges [1]. Its past success was largely driven by the influx of Chinese tourists, which played a pivotal role in the industry's considerable growth. However, recent substantial decline in Chinese and other key markets, during and after the pandemic has highlighted the vulnerabilities and imbalances within Thailand's international market mix. Additionally, the industry faces pressing challenges related to sustainability and environmental concerns. In response, the Thai tourism sector is now shifting its focus toward high-value, technology and cultural driven international markets to address these issues and ensure long-term resilience and sustainable tourism. New travel experiences including gastronomy and local products are key focal points of the marketing approach, driving growth in both short-haul and long-haul international markets, as highlighted by Thailand tourism authority [2].

According to statistical data from the Thailand Ministry of Tourism & Sports [3], data and trends from pre and post the pandemic periods indicate numerous potential markets for Thailand that remain underexplored. Notably, the American tourist market in Thailand, one of the key expanding long haul international markets [2], has demonstrated a significant upward trend in both visitor numbers and spending. Between 2015 and 2019, the number of American tourists visiting Thailand increased by 34.62 %, surpassing one million visitors. According to a study by the Tourism Council of Thailand [4], American tourists have an average stay duration of 15 days per trip in Thailand. Their total spending per stay amounts to THB 96,296, which exceeds the expenditures of tourists from Chinese, European, and other Asian markets.

Furthermore, trend reports underscores Thai tourism industry's potential with the millennial generation which currently travels longer and spends more than other generations including the younger generation, Gen Z [5,6]. The cohort's travel preferences reflect open-minded attitudes, mindful spending, and a desire for meaningful and sustainable experiences [7,8]. According to VISA [9], millennials made up 51 % of international visitors to Thailand in 2023, and this group is expected to further increase spending on experiential travel [7].

To effectively engage millennial travelers, tourism marketing organizations must carefully analyze psychological insights relating to destination selection processes, which is critical for developing tailored communication that resonate with their preferences and values [10]. The millennials cohort exhibits enduring preferences, values, and beliefs that significantly influence their consumption and travel behaviors [11]. Known for their technological proficiency, millennials actively engage online by sharing experiences, participating in travel discussions, and emphasizing experiential travel as a means of conveying social status [12].

The rise of social networks as primary communication channels has significantly influenced millennials' choice of travel destinations, particularly during and after the global pandemic [13,14]. Millennials are strongly influenced by content shared by others on these platforms [15]. They often following trends that amplify marketing efforts, exhibiting a herd mentality [16,17]. Millennials’ strong sense of social connectivity, their tendency to engage in social interaction, and their inclination to conform to the lifestyles of significant others shape their perception of social value [18]. As a result, these digital interactions create and deepen the desire to visit certain destinations.

Consequently, according to Armutcu, Tan [19], the destination choices of millennials, influenced by these factors, present the opportunities to gain deeper insights into psychological needs and expectations of the cohort. Understanding millennials' common psychological perspectives is essential for crafting effective destination marketing strategies. For millennials, the allure of travel is rooted in the anticipation of distinctive and enriching experiences that foster personal development [20].

The influence of peers, fostered through continual social media interactions and perceived social return, notably indicates the bandwagon effect [21]. The unique consumption and travel behavior exhibiting the so-called social media-induced tourism [20]. Millennials' expanded peer networks, which include connections across various media platforms, expose them to vast amounts of information. Consequently, they rely on various cues to filter through this abundance of information from diverse sources. They also share their own travel experiences with the expectation of receiving validation through likes, loves, or comments, thus integrating these experiences into their social identities [15].

As a result, socially conscious millennials often base their travel decisions on the perceived value of others’ behaviors, aiming to create an idealized self-image [20,22]. The pervasive influence of social interaction significantly shapes millennials' travel dispositions, making it a pivotal reference in their decision-making process [23].This suggests that millennials' travel choices may be influenced by heuristic and cognitive biases where observing others' amplified travel behaviors on social media determine their decisions.

Therefore, an updated approach is necessary, integrating new psychological insights from economic theory to effectively illuminate their decision-making processes. This study aims to explore how heuristic and cognitive biases, specifically the bandwagon effect, influences the intention of American Millennials to visit distant destinations such as Thailand in the aftermath of the global pandemic. The bandwagon effect, defined as the propensity for individuals to adopt and imitate certain beliefs and behaviors of others [24], is analyzed within the framework of the theory of planned behavior (TPB).

This research introduces behavioral economic concepts into the study of tourism decision-making, an area of significant importance that has not been extensively investigated [25]. Applying economic theories in empirical research can enhance our understanding of tourist behavior [25]. Behavioral economics, in particular, increases explanatory power by incorporating realistic psychological aspects while still acknowledging the principles of neoclassical economic theory [26].

Travelers, even those who are well-informed do not always behave rationally and can be influenced by various biases and external factors [27]. Their travel-related consumption often prioritize pleasure-seeking over utility maximization [28]. Consequently, the traditional paradigm of rational decision-making, which assumes that travelers make decisions rationally, may not be universally applicable.

An alternative, the dynamic dual-process model that integrates affect-driven heuristic processes with rational logical processes has been proposed as an alternative [29]. Recent tourism studies have introduced various mechanisms and frameworks of dual-process theory [30,31]. The theory suggests that decision-making involves two interrelated and complementary processes [32], where distinct concepts coexist to arrive at a unified decision [33]. One framework posits that travelers utilize both logical-analytic processes and heuristically motivated factors when making decisions, particularly within the social media environment [31,34,35].

The commonly used general decision-making model based on rational and logical processes in travel and tourism is the theory of planned behavior (TPB). The theory offers a viable and proven framework that effectively incorporates various dimensions of human behavior, enabling the analysis and prediction of factors influencing tourists' decision-making [36]. This is especially pertinent in situations where individuals must choose between alternatives, such as selecting a travel destination [37].

Despite its widespread application, the TPB has been criticized for its limitations in fully elucidating individuals' behavioral intentions [38]. Recent studies by Bui [39], Lee and Hwang [40], and Soliman [41] suggest that extending the TPB model enhances its predictive and exploratory power, particularly regarding destination selection. The conventional approaches often overlooks and inadequately considers the social context and the dynamic interactions between individuals [42]. Moreover, while TPB's antecedents explain the reasons for intention, they do not account for explicit motivation [43]. Recognizing the importance of mental processes, including emotions and motivations, is essential for comprehending decision-making mechanisms and predicting their destination intention accurately [29].

The TPB is proposed as a foundational framework linking psychological factors to the selection of travel destinations [44]. To enhance its explanatory power and offer more behaviorally realistic of the decision-making process, researchers have incorporated additional cognitive psychology variables into the traditional model [41,45,46]. This study adopts a similar approach by integrating the bandwagon effect into the context of travel intention, aiming to gain deeper insights into travelers' behaviors and improve the effectiveness of tourism strategies that address inherent biases [20]. While extensively studied in consumer behavior, the bandwagon effect's application to tourism remains underexplored. This research introduces a multi-dimensional perspective on the bandwagon effect, incorporating heuristic and cognitive biases from behavioral economics into the traditional TPB framework. The study proposes attitude and subjective norm, key volitional determinants that motivate engagement in behaviors [37], as mediators. The proposed framework aims to enhance understanding of millennial travelers' decision-making by examining the dual-process concept, where a rational, logical approach coexists with an intuitive, preference-driven approach in destination selection.

## 2 Literature review

### 2.1 Theory of planned behavior

The theory of planned behavior (TPB) has been extensively used in the field of tourism, especially in predicting travel-related behaviors since it was proposed by Ajzen and Driver [47]. The predictors of the TPB are attitude, subjective norm, and perceived behavioral control [48,49]. According to the theory, individuals’ behavioral intention emerges as the principal factor determining proximal action or behavior [50] and express intention consistent with their attitude and subjective norm [51]. The interrelation between individuals' beliefs about the anticipated outcomes, beliefs about societal expectations regarding what to do, and enhance by beliefs about the capability to execute the behavior predicates the formation of a behavioral intention [48]. In the realm of travel and tourism, behavioral intention denotes the probability that a prospective traveler will undertake a visit to a particular place or destination [52].

The study proposes employing attitude and subjective norm as mediating factors within a framework that emphasizes volitional process with socially significance [53]. Attitude and subjective norm, which are original antecedents of behavioral intention from the theory of reasoned action are categorized as volitional components [37,49]. These components represent the central motivation behind behavior intention. Furthermore, when behaviors are performed to achieve specific goals, attitudes and subjective norms, as indicators of perceived consequences and social approval, they become key determinants toward subjectively valued outcomes [54]. In cases where a study focuses on intention toward socially valued, goal-oriented behavior, or when individuals are confident in their ability to execute the desired behavior, a non-volitional component such as perceived behavioral control becomes less relevant [37].

On the other hand, it is suggested that perceived behavior control may exert influence on behavior rather than intention, primarily due to the limited knowledge individuals possess about actual behavioral control [54]. Furthermore, recent studies also postulates a perceived behavior control role as moderator instead of direct influence toward intention as it was originally proposed [37,51,55].

Individuals' evaluation of behavior or the tendency to react positively or negatively to a particular behavior defines attitude [48]. It is determined by his or her accessible beliefs about the behavior of interest. Specifically, attitude toward focused behavior is presumed to be determined by readily accessible beliefs or expectancies concerning the likely outcomes [54]. Attitude is thought to arise through education, observations, and experiences which individuals respond to over time. When a person is exposed to or thinks about an action, their formulated attitude, based on readily accessible beliefs, is automatically evaluated [37]. In the context of destination choice, attitude toward traveling behavior refers to individuals’ collection of knowledge, beliefs, and feelings toward traveling behavior involving expected outcomes of visiting a destination of interest.

In a socially connected society, the subjective norm refers to individuals' belief that their broader social network approves of and supports their behavior, prompting them to engage in the behavior based on these perceived social expectations [56]. The subjective norm in the TPB pertains to normative beliefs regarding specific social referents that hold personal significance to the individuals. It examines how these readily accessible beliefs generate perceived social endorsement, thereby influencing whether they will engage or refrain from a particular behavior [54]. According to Ajzen [37] injunctive normative beliefs, which reflect approval or disapproval from relevant others, and descriptive normative beliefs, which reflect the likelihood of important others performing the behavior themselves, both exert significant influence when considering whether to perform a specific behavior.

Attitude and subjective norm have consistently been confirmed as key determinants of behavior when analyzing the intention to visit. Travelers’ subjective assessment of outcomes and perceived endorsements of travel-related behaviors are critical in determining their intention to visit. Recent studies reaffirm that both attitude and subjective norms influence the visiting intentions of American travelers [38,53,57]. Moreover, recent research further supports the influence of attitude and subjective norm on bandwagon-driven consumption behaviors [21,58]. Consequently, this study hypothesizes the structural relationships that represent the impact of attitude and subjective norm on the intention to visit Thailand.

H1 Attitude positively influences intention to visit.

H2 Subjective norm positively influences intention to visit.

Recent models of TPB propose an interrelationship among the core predictors of the TPB [37], specifically highlighting the positive impact of subjective norm on attitude [53]. This connection is rooted in the notion that, while individuals are the ultimate decision-makers for specific behaviors, the opinions of significant others can shape their attitude toward these behaviors [49]. Additionally, it is suggested that normative factors, such as subjective norm, are positively correlated with attitudinal factors, thereby enhancing the simplicity and explanatory power of the framework [53]. Based on these insights, this study hypothesizes a structural relationship that represents the impact of subjective norm on attitude toward traveling to Thailand.

H3 Subjective norm positively influences attitude.

### *2.2* *Behavioral economics*, *heuristics and cognitive biases*

An alternative approach suggests integrating psychological insights and economic aspects into decision-making frameworks to gain a deeper understanding of human behavior. The basic principle of the traditional decision-making approach dictates that individuals make rational choices, aiming to achieve the optimal outcome [29,59]. In reality, bounded rationality or limitation of rationality occurs when decisions are made under cognitive limitations that constrain individuals' evaluation abilities [60]. Bounded rationality, combined with heuristics and biases, elucidates a suboptimal process that affects decision-making in various contexts [26,61].

When individuals selectively allocate attention based on circumstances and surrounding events, it can affect outcomes [60,62]. In complex situations, individuals overcome obstacles like information overload by using cognitive shortcuts or heuristics [63]. Conversely, efficient decision-making often relies on utilizing established information. Biased decisions are typically made intuitively, influenced by limited data and social context [60,64]. Individuals frequently face cognitive demands, beginning with attention processes [59] and extending to the evaluation of resources, integration of information, and thorough examination of alternatives. Consequently, individuals frequently use heuristics and cognitive biases to streamline their efforts for specific tasks associated with decision-making processes [65].

Cognitive bias psychology offers valuable insights into individual behaviors, particularly in leisure-related contexts. It is especially pertinent when evaluating the efficacy of communication aimed at intended travelers [66]. Psychologists suggest that many of these biases and heuristics serve as an adaptive instrument, accelerating decision speed and enhancing decision confidence [67]. Exploring the possible relation between cognitive preferences and behaviors is vital to understand the effectiveness of communication strategies in different contexts of travelers' decision-making processes [29].

In present-day social media influences travelers who heavily rely on online information, such as reviews, ratings, and recommendations, indicating the presence of cognitive biases in their evaluation of destination choices [20]. Utilizing low-involvement methods to process and interpret information is often necessary, as it can expedite decision-making process and make it more economical [63]. The cues or shortcuts in this heuristic-influenced process require less effort due to the easily discernible cues. In combination, these heuristics and biases lead travelers to systematically make suboptimal choices based on intuition-driven preferences [68].

### 2.3 Bandwagon effect

Social psychology defines a term for the tendency of people to align their beliefs and behaviors to those of a reference group for the incentive of social inclusion and perceived social return as herd mentality or bandwagon bias [21]. This cognitive psychology mechanisms phenomenon can emerge both consciously and unconsciously during the process of information handling [69]. Individuals' inclination toward social interaction and conformity is encouraged by significant others, leading to a magnified effect across the social group [18,24]. This cognitive bias pertains to the tendency of individuals to conform to the consensus established by the collective judgement of the community [70], even when it differs from their own intrinsic needs or beliefs [71,72]. In the social networking information society, highly engaged individuals are more likely to integrate input from their social circle into their decision-making processes due to a higher level of trust in relevant others [27] and especially when conforming brings social rewards [71].

In social media-driven consumer behavior research, it's observed that individuals are progressively making purchasing decisions influenced by the collective choices and opinions of their peers and networks. The combined effect of digital collaborative endorsement [73], persuasive power of parasocial relationships with social media influencers [74] and the effectiveness of information dissemination [75] collectively shape consumers' intentions to purchase by leveraging the bandwagon effect [76].

Similarly in tourism, the influence of the bandwagon effect on travelers' decisions has notably increased as online platforms have become essential channels for sharing travel-related information and experiences [69], impacting both their engagement in travel activities and their selection of destinations [77]. Travelers repeatedly convey their trips to signal their personality and status, and those who constantly connect within their social circles acquire insights into the travel behaviors of their peers, potentially fostering the formation of group preferences through subjective evaluation of destinations [24].

For selecting tourism destinations, the bandwagon effect is an occurrence in which a large group of travelers chooses certain destinations, often disregarding their own intrinsic needs or preferences [78]. This phenomenon is amplified by the need for social connectedness and the corresponding symbolic value [79]. During the process of destination selection, from singling out attention toward a few choices to evaluating those chosen destinations, the bandwagon effect inserts its influence on individuals along the process, as identified, explained, and summarized by Refs. [24,78,80,81]. In conclusion, the bandwagon effect in the travel destination context reflects the tendency or inclination toward certain destinations to satisfy extrinsic needs of social validation.

Nadroo, Lim [76] provides an overview of various operationalizations of the bandwagon effect in the consumer-related field. Currie, Wesley [82] classifies several effects of the bandwagon, including informational, utilitarian, value-expressive, and normative influences. In the context of tourism, Boto-García and Baños-Pino [24] identify three mechanisms of the bandwagon effect: conspicuous consumption, normative influence, and informational influence. Furthermore, Korteling and Toet [64] delineate the cognitive bias psychology frameworks into capacity-based and expertise-based. Within these frameworks, three psychological domains of the bandwagon effect emerge: bandwagon cue, normative influence, and informational influence.

Bandwagon cue refers to the social media complied information indicating societal endorsed behavior and content [83]. Bandwagon cue such as number of likes and comments represent the level of interaction is an important heuristic element used in evaluating essential information for subsequent decision-making processes leading to decisions and behaviors [84]. Millennials, who heavily rely on online media, actively seek to identify, and utilize cues dispensed on the platform to enhance their information processing and decision-making abilities [85]. Tourists heuristically process data based on its accessibility, including non-textual signals and characteristics of sources [86]. A systematic shortcut allows tourists to assess, expand, and acknowledge the content of the message. Bandwagon cues enhance the credibility of content and messages, while also exerting strong influential power on decision-making [70]. The interpretation of this operation is biased depending on the asserted level of influence and legitimacy of the message and messenger [32]. The heuristic visual cues represented by platform rating indicators potentially impact travelers’ attitudes and intentions [87].

Normative influence refers to the socially motivated tendency of individuals' thoughts, feelings, and behaviors to conform to the needs for acceptance and belonging within a group [88]. Normative influence arises when the behavior observed in relevant others is seen as a benchmark for what is considered acceptable conduct [89]. It involves a shift in individuals' evaluation of products or services due to exposure to the assessments, intentions, or purchasing behaviors of others [90]. Participation and interaction between members of social groups, including people from social media, enable the learning of others' travel behaviors [91] and affect individuals’ subjective preferences and evaluation of destinations. The motivation to travel for the reward of conformity and convey status lifestyle [18] represents the normative influence framework compliance with certain peer groups.

Informational influence refers to individuals’ inclination to accept pertinent information from others as valid, factual or indicative of reality [27,88]. Social platforms simplify interactions between content creators and travelers, leading travelers to assign greater credibility to the information they have obtained [18]. Travelers exhibit pragmatism regarding their preferred information sources about the destination during the initial stage, potentially formulating their intended behavior [92]. Researchers have observed that frequent travelers actively seek information and validation through their social networks, strongly influencing their travel-related decisions. The role of cognitive limitations is significant as it facilitates tourists' utilization and convenient access to information, particularly prior to their trips [92]. Travelers often engage in information-seeking behaviors and rely on recommendations regarding accommodations, attractions, and activities [93], with social media platforms being crucial in their decision-making process. Informational influence explains the internalization process to conform to the opinions of others because they want to be correct and believe that others may have better knowledge or understanding of the destinations [82]. When the influence of other travelers is presented, it endorses and increases the tendency for individuals to believe the information and its sources [94].

For a profound understanding of the underlying process, the multidimensional bandwagon effect encompassing bandwagon cue, normative influence, and informational influence is essential to explain individuals' elemental biases behind travel-related decisions. Decision-making models should include psychological processes that recognize cognitive biases and heuristics as determinants in destination selection [29]. Several studies confirm positive relationships between bandwagon-related factors and behavioral intention [45,79,81,95,96].

Observing others' successful experiences can inspire and influence travelers, especially in times of uncertainty [24,97]. Moreover, persuasive influences from relevant others can significantly affect destination perception, perceived values, and social return [21,77,79]. Similarly, the need to identify and conform with social groups also plays a significant role in shaping behavioral intentions, as emphasized by Beall, Boley [80] and Wasaya, Prentice [89]. Therefore, the bandwagon effect's direct relation to behavioral intention, particularly from millennials' perspectives [98,99], could offer a more behaviorally realistic understanding of the intuitively-driven decision-making process. This study hypothesizes a structural relationship that represents the impact of the bandwagon effect on the intention to visit Thailand.

H4 Bandwagon effect positively influences intention to visit.

Attitude and subjective norm are often emphasized [95] and shown to be significantly influenced in studies where the bandwagon or related factors are concerned [45,79]. The volitional components of the theory of planned behavior are based on individuals' behavioral and normative beliefs, and the bandwagon effect has the potential to shape and alter these beliefs throughout the decision-making process. This aspect is particularly significant in meeting the psychological needs of socially conscious individuals.

Attitude toward a focused behavior is influenced by the bandwagon effect [72], which amplifies the perceived usefulness, expected utility and enhances the level of self-assured judgment [21,27,100], particularly, when there is limited actual experience [101]. Furthermore, a positive impact on subjective norm arises as social amplification influences individuals' beliefs regarding the social judgment to perform the behavior [102]. As a result, this study hypothesizes the structural relationships that represent the impact of the bandwagon effect on attitude and subjective norm toward traveling to Thailand.

H5 Bandwagon effect positively influences attitude.

H6 Bandwagon effect positively influences subjective norm.

### 2.4 The mediating roles of attitude and subjective norm

Although the bandwagon effect within the framework of the extended TPB has not been extensively studied in tourism-related studies, the mediating role of attitude and subjective norm can be suggested. Attitude and subjective norm are validated to mediate the effect of affective and socially induced antecedents toward travel intention [46,103,104]. Several studies intuitively support the mediating role of attitude and subjective norm in the context of the bandwagon effect or related factors influencing behavioral intention [79,95,101].

The assumption arises because decision-making is a process where the bandwagon effect, or the tendency to mimic others’ behavior, can influence individuals' internal decision-making processes via various mediators before arriving at behavioral intention. Individuals' beliefs form the foundation of each predictor in the TPB, and these beliefs can be influenced during the decision-making process [32] by external heuristic and cognitive bias factors such as the bandwagon. Based on the insights from related studies, this study hypothesizes the structural relationships that represent the mediating roles of attitude and subjective norm.

H7 The relationship between bandwagon effect and intention to visit is mediated by attitude.

H8 The relationship between bandwagon effect and intention to visit is mediated by subjective norm.

H9 The relationship between bandwagon effect and intention to visit is mediated by attitude and subjective norm.

**Fig. 1:**
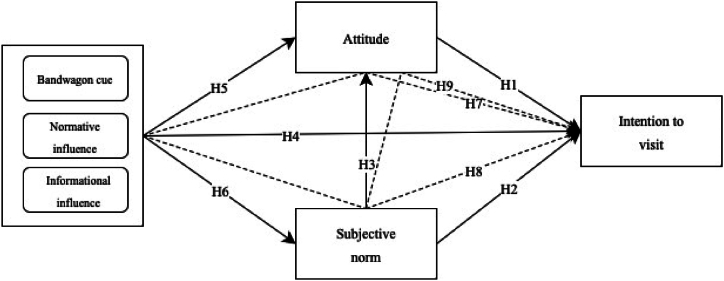
Conceptual framework.

Fig. 1 Alt text. Conceptual framework descripting bandwagon effect with sup-dimensions effecting intention to visit directly and indirectly via attitude and subjective norm.

## 3 Methodology

### 3.1 Sampling and data collection

The study aimed to investigate the impact of the bandwagon effect, attitude, and subjective norm on the intention to visit Thailand for the first time among American millennials. A multistage sampling technique was employed, with the first stage utilizing quota sampling applied to 10 selected large metropolitan statistical areas in the United States with the highest proportion and proportion increase of millennials, according to United State Census Bereau [105].

The following metropolitan statistical areas were targeted as sampling locations: Austin-Round Rock-Georgetown, Denver-Aurora-Lakewood, Orlando-Kissimmee-Sanford, San Francisco-Oakland-Berkeley, Seattle-Tacoma-Bellevue, Dallas-Fort Worth-Arlington, Los Angeles-Long Beach-Anaheim, Houston-The Woodlands-Sugar Land, Nashville-Davidson-Murfreesboro-Franklin, and Washington-Arlington-Alexandria.

The second stage employed convenient sampling with specific inclusion criteria, utilizing online platforms to gather surveys for hypothesis testing in the exploratory phase [106]. The target respondents were prospective millennial visitors to Thailand, a demographic that aligns with strategic priorities in Thailand's tourism sector [2]. The survey population comprises digitally dependent, higher-income millennials who actively use social media in making travel-related decisions and are planning their first visit to Thailand. The inclusion criteria were as follows: 1) American residents aged 27–42) middle-to upper-income levels, 3) no previous visits to Thailand, and 4) frequently seen Thailand-related travel content on social media. The sample selection occurred between September and October 2023, resulting in 358 completed responses and a response rate of 56.37 %.

### 3.2 Measurement instrument development

The questionnaires were designed in American English to suit the research's focus on the American population, following Ajzen and Driver [47] recommendation for measurement model incorporation of seven-point Likert scales and seven-point semantic differential scales. This aligns with the researcher's adherence to the TPB. The bandwagon effect encompasses bandwagon cue dimension [85,86], along with normative and informational influences [91,107]. Measurement items for the bandwagon cue dimension were derived from Kim, Brubaker [108] study. Items for the normative and informational influence dimensions were adapted from scales developed by Book and Tanford [91], which were based on Deutsch and Gerard [109] research.

Bipolar semantic differential scales were utilized to assess the level of attitude, as proposed by Ajzen and Driver [47]. Participants were instructed to evaluate Thailand as a travel destination. The choice of measurement items for attitude has shown variability across previous studies within the context of travel destinations. In this study, the initial three measurement items were drawn from recent research [38,53,110,111], grounded in the framework provided by Ajzen and Driver [47]. Additionally, three supplementary measurement items specific to Thailand were developed based on information gathered from travel-related web articles [[112], [113], [114], [115]].

Measurements of subjective norm and intention to visit have been widely utilized since they were first proposed by Ajzen and Driver Ajzen [116] and have appeared in numerous past and recent studies focusing on destination selection [41,53,110,117]. Measurement items for subjective norm and intention to visit were derived from these prior studies.

Exploratory factor analysis was initially employed with a sample size of 100 to verify the factor structure and validate measurement items related to the constructs of the bandwagon effect and attitude. These measurements were not directly derived from previous studies nor had substantial support. The analysis revealed three rotated components for the bandwagon effect and one rotated component for attitude. Validation criteria included factor loadings above 0.55 (ranging from 0.620 to 0.904), Eigenvalues surpassing 1 (ranging from 2.469 to 3.885), and cumulative percent of variance exceeding 50 % (ranging from 64.75 % to 79.37 %). The Kaiser-Meyer-Olkin (KMO) measure ranged from 0.872 to 0.838, indicating sampling adequacy. Additionally, Bartlett's test was significant (p < 0.001), meeting the prerequisite assumptions for factor analysis.

Partial Least Squares Structural Equation Model (PLS-SEM) with a series of analyses, as suggested by Hair Jr, Hult [118] was used to analyze multivariate data and simultaneously assess a series of multiple regressions as well as the structural model. PLS-SEM is suitable for explanatory and predictive analysis on a complex hypothetical model with low theoretical endorsement and small sample sizes [119]. Additionally, it is a preferred method to use when variables within the model exhibit significant non-normalized data [118]. PLS-SEM has been used in several studies concerning an extended theory of planned behavior investigating behavioral intention [40,41,111].

## 4 Results

### 4.1 Socio-demographic profiles

Table 1 shows that among the 358 respondents, 52.20 % were female and 47.80 % were male. In terms of marital status, 50.80 % were partnered with children, 24.60 % were partnered without children, and 18.40 % were single. Among those respondents, 50.80 % had a college degree, and 40.20 % had an advanced degree. 80.40 % of respondents were full-time employees. The annual household income of respondents was distributed as follows: 54.20 % were classified as lower-middle-income, and 31.60 % as upper-middle-income.

**Table 1:** Respondents’ sociodemographic profiles.

Demographic	Categories	Frequency	Percentage
Gender	1. Male	171	47.80 %
	2. Female	187	52.20 %
Marital status	1. Single	66	18.40 %
	2. With partner with children	182	50.8 %
	3. With partner no children	88	24.60 %
	4. Separated/Divorced/Widowed	17	4.70 %
	5. Prefer not to answer	5	1.40 %
Education level	1. High School	29	8.1 %
	2. Collage degree	182	50.8 %
	3. Advanced degree	144	40.2 %
	4. Prefer not to answer	3	0.8 %
Occupation	1. Unemployed	26	7.3 %
	2. Entrepreneur	14	4.2 %
	3.Full time employee	284	80.4 %
	4. Part time employee	20	5.9 %
	5. Retired	3	0.8 %
	6. Prefer not to answer	5	1.4 %
Annual household income	1.USD 50,000–99,999	194	54.2 %
	2. USD 100,000–149,999	113	31.6 %
	5. USD 150,000 and above	51	14.2 %

To assess sampling bias, we compared the sample's gender, marital status and education profiles with population data from the United States Census Bereau [120]. The sample's gender profile is relatively close to that of the population, suggesting minimal gender bias. However, the sample differs significantly in other areas, with an overrepresentation of married individuals and those with a college education or advanced degree.

### 4.2 Measurement model assessment

Confirmatory Composite Analysis (PLS-CCA) was employed to identify three key dimensions: bandwagon cues, normative influence, and informational influence as well as validate the composite measurement model of the constructs. PLS-CCA employing Construct Reliability and Validity, was assessed utilizing Cronbach's Alpha Coefficient, Composite Reliability rho_a (CR), and Average Variance Extracted (AVE) as guidelines by Hair Jr, Hult [118].

As shown in Table 2, Cronbach's Alpha Coefficient ranged from 0.775 to 0.913, with CR scores exceeding 0.70 (0.790–0.913). Together with AVE scores exceeding 0.50 (0.630–0.793), the reliability and validity of the measurement model were satisfied. Moreover, the measurement items mostly exhibited significant t-statistics with outer loading exceeding the threshold of 0.708. One item from attitude construct loading score was lower than the threshold. However, the item was retained as its removal would not increase internal consistency reliability [118].

**Table 2:** Confirmatory composite analysis.

Variables	Measurements	Outer loading	t-statistic	Cronbach Alpha	CR	AVE
Bandwagon cue				0.913	0.913	0.793
	I'm likely to be interested in social media contents about traveling to Thailand because the popularity of the content as represented by level of content engagement.	0.884	62.024∗∗∗			
	I'm likely to be interested in social media contents about traveling to Thailand because the popularity of the content creator as represented by number of subscribers & followers.	0.914	82.996∗∗∗			
	I'm likely to be interested in social media contents about traveling to Thailand because the topic as current popular and emerging as trend.	0.879	61.198∗∗∗			
	I'm likely to be interested in social media contents about traveling to Thailand because the popularity of the social media platform.	0.884	60.003∗∗∗			
Normative influence				0.913	0.851	0.793
	I'm interested in traveling to Thailand after seeing others' experiences of visiting on social media because following their actions is likely to result in my inclusion within social group.	0.882	65.991∗∗∗			
	I'm interested in traveling to Thailand after seeing others' experiences of visiting on social media because following their actions is likely to result in my visibility within social group.	0.925	119.628∗∗∗			
	I'm interested in traveling to Thailand after seeing others' experiences of visiting on social media because I feel pressured to follow others.	0.797	34.497∗∗∗			
Informational Influence				0.887	0.892	0.746
	Social media contents are an important source of information about traveling to Thailand because I believe that information to be credible.	0.850	57.711∗∗∗			
	Social media contents are an important source of information about traveling to Thailand because People who have visited have more knowledge than I do.	0.853	41.511∗∗∗			
	Social media contents are an important source of information about traveling to Thailand because I think information helps me deciding between choices	0.869	49.433∗∗∗			
	Social media contents are an important source of information about traveling to Thailand because I value opinions and recommendations of others	0.883	60.477∗∗∗			
Attitude				0.880	0.886	0.630
	All things considered; I personally think traveling to Thailand would be enjoyable.	0.859	38.630∗∗∗			
	All things considered; I personally think traveling to Thailand would be entertaining.	0.871	49.756∗∗∗			
	All things considered; I personally think traveling to Thailand would be adventurous.	0.760	26.648∗∗∗			
	For me, a trip to Thailand would be value for money.	0.805	29.893∗∗∗			
	For me, a trip to Thailand would be authentic.	0.787	27.839∗∗∗			
	For me, a trip to Thailand would be serene.	0.661	19.758∗∗∗			
Subjective norm				0.775	0.790	0.686
	People who are relevant to me think I should visit Thailand.	0.835	49.500∗∗∗			
	People who are relevant to me would approve of me visiting Thailand.	0.825	36.196∗∗∗			
	People who are relevant to me would visit Thailand themselves.	0.825	31.454∗∗∗			
Intention to visit				0.863	0.864	0.786
	Thailand would be among my first choice of destinations.	0.857	52.221∗∗∗			
	I will visit Thailand in the future.	0.890	69.428∗∗∗			
	I will save time and money for the purpose of visiting Thailand.	0.911	85.599∗∗∗			

Combining the results from the Fornell-Larcker Criterion and Heterotrait-Monotrait ratio (HTMT) ratios as shown in Table 3, the discriminant validity of the model was satisfactory. The measurement model demonstrated the Fornell-Larcker criterion, as the square root of AVE for all the constructs were higher than the correlation between constructs. Additionally, HTMT ratio of correlations did not exceed the threshold of 0.85 [118].

**Table 3:** Discriminant validity analyses.

	Bandwagon effect	Attitude	Subjective norm	Intention to visit
**Fornell-Larcker**				
Bandwagon effect	**0.755**			
Attitude	0.494	**0.793**		
Subjective norm	0.525	0.636	**0.828**	
Intention to visit	0.624	0.679	0.672	**0.886**
**HTMT**				
Bandwagon effect				
Attitude	0.541			
Subjective norm	0.601	0.764		
Intention to visit	0.698	0.769	0.802	

### 4.3 Structural model assessment

The structural model assessment elucidates the relationships between the bandwagon effect, attitude, subjective norm, and intention to visit, demonstrating direct and indirect effect within the framework. Firstly, the multicollinearity issue was evaluated by the Variance Inflation Factors (VIF). The model exhibited VIF values lower than the 3.3 threshold; therefore, the study concludes that there was no issue of multicollinearity in the structural model [121].

Then, the parallel processing option employing 10,000 bootstrap resampling was executed as recommended by Hair Jr, Hult [118] to test the significance level of path coefficients. The path coefficient measures the relation strength and sign of effect in the regression model, with a p-value less than 0.05 indicating the statistical significance of the path as recommended by Kock and Lynn [121].

The proposed hypotheses were all significantly supported with a series of indicators, including path coefficients, p-value, and coefficients of determination (R^2^), to explain and assess the hypothetical model. According to the structural relation results in Table 4, attitude positively influences intention to visit (β = 0.341, p < 0.001), subjective norm positively influences intention to visit (β = 0.298, p < 0.001) and subjective norm positively influences attitude (β = 0.520, p < 0.001).

**Table 4:** Structural model assessment.

Hypotheses	Structural Relation	β	t-statistic	p-value	VIF
H1	Attitude - > Intention to visit	0.341∗∗∗	6.280	<0.001	1.787
H2	Subjective norm - > Intention to visit	0.298∗∗∗	4.865	<0.001	1.865
H3	Subjective norm - > Attitude	0.520∗∗∗	8.286	<0.001	1.381
H4	Bandwagon effect - > Intention to visit	0.299∗∗∗	6.637	<0.001	1.468
H5	Bandwagon effect- > Attitude	0.221∗∗∗	3.867	<0.001	1.381
H6	Bandwagon effect - > Subjective norm	0.525∗∗∗	10.515	<0.001	1.000
H7	Bandwagon effect - > Attitude - > Intention to visit	0.075∗∗	2.963	0.003	
H8	Bandwagon effect - > Subjective norm - > Intention to visit	0.157∗∗∗	4.287	<0.001	
H19	Bandwagon effect - > Subjective norm - > Attitude - > Intention to visit	0.093∗∗∗	4.985	<0.001	

Note: ∗∗∗p < 0.001, ∗∗p < 0.01, ∗p < 0.05.

Furthermore, the bandwagon effect positively influences intention to visit (β = 0.299, p < 0.001), positively influences attitude (β = 0.221, p < 0.001) and positively influences subjective norm (β = 0.525, p < 0.001). In terms of mediation, the relationship between the bandwagon effect and intention to visit is partially mediated by attitude (β = 0.075, p = 0.003) and by subjective norm (β = 0.157, p < 0.001). Additionally, the relationship between the bandwagon effect and intention to visit is also partially mediated by both attitude and subjective norm combined (β = 0.093, p < 0.001).

The study employed the coefficient of determination (R^2^) to evaluate the explanatory capacity of a model. Furthermore, it is recommended to assess the model fit in analyzing human attitudes, perceptions, and intentions [122]. The exogenous factors effectively explained the variance in the endogenous factors. R^2^ Attitude = 0.440, R^2^ Subjective norm = 0.276, R^2^ Intention to visit = 0.619.

**Fig. 2:**
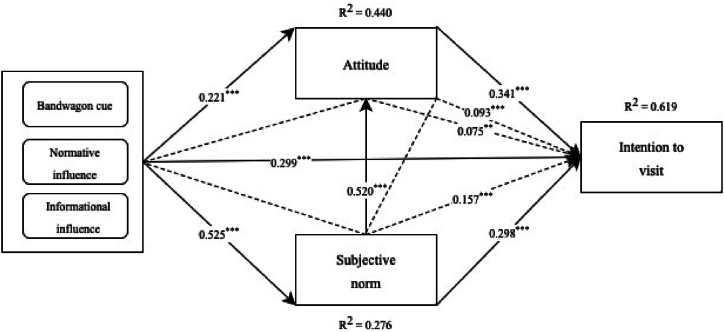
Structural model. ∗∗∗p < 0.001, ∗∗p < 0.01, ∗p < 0.05

Fig. 2 Alt text. Structural model showing the path coefficients and R^2^ of the direct and indirect relation between bandwagon effect, attitude, subjective norm and intention to visit.

The final step in the structural model assessment was to evaluate the predictive power of the hypothetical model. According to Hair Jr, Hult [118], Q^2^ values should be positive values higher than zero, and CVPAT values, which demonstrate average loss value when compared to the benchmark, should be negative values. The results from the model shown in Table 5 demonstrated positive Q^2^ values as well as exhibiting negative average loss values with a p-value <0.001. Therefore, the conclusion can be made that the hypothetical model significantly demonstrates predictive relevance.

**Table 5:** Predictive relevance.

	Q^2^	Average loss difference	t-statistic	p-value
Bandwagon cue	0.828	−1.590	12.132	<0.001
Normative influence	0.654	−1.789	11.674	<0.001
Informational influence	0.726	−1.067	7.954	<0.001
Attitude	0.239	−0.228	4.725	<0.001
Subjective norm	0.269	−0.344	4.454	<0.001
Intention to visit	0.386	−0.693	6.681	<0.001
Overall		−0.890	12.532	<0.001

## 5 Discussion

The study explores the multidimensional nature of the bandwagon effect through the theory of planned behavior, focusing on attitude and subjective norms as mediators in American millennials' intentions to visit Thailand. The findings validate the proposed multidimensional mechanism of the bandwagon effect, in contrast to Nadroo, Lim [76], which suggests cause and effect relationships among different bandwagon-related components. Furthermore, the structural model reveals how this effect influences travel intentions within the TPB framework.

The structural results support Hypotheses H1 and H2, reaffirming that attitude and subjective norm positively influence American millennials' intention to visit Thailand. Specifically, their positive beliefs about the anticipated outcomes and societal expectations increase the likelihood that American millennial travelers to undertake a visit to Thailand in the near future is consistent with recent research [38,40,53]. Additionally, Hypothesis H3 confirms the interrelationship between attitude and subjective norm, demonstrating that normative factors, such as subjective norm, have a positive impact on the attitudinal factors among American millennials align with study by Han, Al-Ansi [53]. As suggested by Wang, Yeh [49], the ultimate decision for their action lies with the individuals and their attitude toward the intended behavior.

The direct impact of the multidimensional bandwagon effect on all other factors, as investigated in Hypotheses H4 through H6, underscores that American millennials' intention as well as attitude and subjective norm are significantly influenced by the behaviors and choices of relevant others within their social networks. The study confirms the significant direct impact of the bandwagon effect on the intention to visit, consistent with post-pandemic findings that emphasize the strong influence of others' opinions on both consumption behavior [22] and tourism destination choice [17]. This outcome underscores the notion that millennial travelers may be influenced to make biased decisions regarding destination choices without always engaging in fully rational decision-making processes.

Additionally, the study elucidates the bandwagon effect's direct impact on key components of the TPB. This aligns with previous research in tourism suggesting that electronic word of mouth and social engagement significantly affect attitude and subjective norm [14,17,39]. These influences confirm that individuals' behavioral and normative beliefs are shaped by the perceived social value as underscored by Boley, Jordan [21] and Han, Wang [107].

The significant impact of bandwagon cues from social media platforms in shaping travel-related decisions, particularly when evaluating the importance of content and its creators, has been highlighted in prior research [39]. The validated measurement model extends beyond content engagement, as studied by Tugores-Ques and Bonilla-Quijada [84] to include the popularity of content, creators, and platforms. Moreover, the confirmed significance of normative and informational influences, as supported by recent tourism studies by Wasaya, Prentice [89] and Han, Wang [107], underscores their significance within the social environment preceding travel decisions [91].

The investigation of Hypotheses H7 through H9 reveals that the bandwagon effect affects travelers’ beliefs during internal decision-making processes, leading to specific behavioral intentions. The partially mediating roles of attitude and subjective norm align with the tourism study of Bui [39] indicating that positive attitude and subjective norm serve as partial mediators, influencing how public support for specific behaviors impacts the intention to act.

The partially mediating roles of attitude and subjective norm also suggest that the bandwagon effect also plays a role in the logical evaluation process, despite variations in cognitive modes [29,32]. Travelers' underlying beliefs form the basis for normative and attitudinal factors, but these beliefs can be influenced by external cognitive biases, highlighting a research gap in the context of tourism destinations, as noted by Goh [101]. Furthermore the result asserts the importance of a causal path between socio-psychological factors leading to behavioral intention [49]**.**

Additionally, the structural relation presented in Table 6 indicates a complex dual process that integrates two decision-related systems [29,31,35,123]. One system utilizes the bandwagon effect as a heuristic and cognitive bias factor, directly influencing intention. Simultaneously, the other system involves systematic evaluation through attitude and subjective norm to determine intentions. The results reveal a dual approach to cognitive information processing, highlighting that heuristic and systematic processes operate concurrently and interdependently to influence decision-making outcomes.

**Table 6:** Results of direct effects and indirect effects.

Structural Relation	Path Coefficient	t-statistic	p-value
**Attitude - > Intention to visit**			
Total effect	0.341	6.280	<0.001
Direct effect	0.341	6.280	<0.001
**Subjective norm - > Intention to visit**			
Total effect	0.476	8.385	<0.001
Direct effect	0.298	4.865	<0.001
Indirect effect	0.177	5.860	<0.001
**Bandwagon effect - > Intention to visit**			
Total effect	0.624	16.845	<0.001
Direct effect	0.299	6.637	<0.001
Indirect effect	0.325	9.790	<0.001

The findings are consistent with the online information processing research conducted by Kwon, Lee [31] and resonate with prior studies by Kock, Josiassen [123] and Lindberg and Stemmer [124] which emphasize that decision components are processed differently in travelers' mental decision-making regarding destination choices. This dual process elucidates how travelers, particularly American millennials, navigate and process information when evaluating travel destinations. This cohort employs both socially constructed preferences and logical evaluations to arrive at a unified decision regarding their preferred destinations.

The multigroup analysis outcomes presented in Table 7 reveal a disparity in the influence of the bandwagon effect on subjective norms, with males showing a stronger response than females. This finding contrasts with the consumer behavior study by Liu [125], though it aligns with research by Croes and Bartels [126], which indicates that males are more likely than females to engage with and be influenced by social media influencers, driven by their extrinsic motivation for social approval.

**Table 7:** Multigroup analysis results.

	Gender Female vs Male	Education College - Highschool	Education Advance - Highschool	Education Advance - College
Attitude - > Intention to visit	0.213	−0.076	0.163	0.24∗
Subjective norm - > Intention to visit	−0.023	0.131	−0.008	−0.139
Subjective norm - > Attitude	−0.045	0.333	0.167	−0.166
Bandwagon effect - > Intention to visit	−0.148	0.094	−0.02	−0.114
Bandwagon effect- > Attitude	−0.167	−0.162	0.14	0.302∗
Bandwagon effect - > Subjective norm	−0.247∗∗	0.065	0.271	0.206∗

Note: ∗∗∗p < 0.001, ∗∗p < 0.01, ∗p < 0.05.

Furthermore, the results indicate that the bandwagon effect has a stronger impact on both attitude and subjective norm among individuals with advanced degrees compared to those with only a high school education. Additionally, the analysis shows a significant difference in how attitude affects the intention to visit, with individuals holding advanced degrees exhibiting a greater influence than those with college degrees. These findings are consistent with the consumer behavior study by Croes and Bartels [126] and Liu [125], which suggests that highly educated individuals are more likely to be influenced by social media influencers to fulfil their need for reliable and practical information.

## 6 Conclusion, implications and limitations

### 6.1 Conclusion

This study advances the tourism sector by providing key insights into the behavior of American millennial travelers. It presents a framework that integrates the bandwagon effect with the key motivational determinants of volitional behavior, particularly focusing on socially significant factors namely, attitude and subjective norms. By incorporating heuristic and cognitive bias factors into traditional theory, this unified dual-process reconceptualization enhances the model's explanatory and predictive capabilities.

The study first highlights the significant influence of attitude on American millennials' intention to visit Thailand. Measurement assessments reveal that these travelers not only seek entertaining and enjoyable experiences but also prioritize value for money. Moreover, subjective norm plays a crucial role in shaping intentions, as the approval of relevant others not only influences destination selection but also impacts the evaluation of anticipated outcomes.

Secondly, the findings underscore that the bandwagon effect significantly influences American millennials, who tend to conform to the travel behaviors of others due to the perceived social values [18,21]. Their intentions are shaped by the collective behavior of others, particularly through relevant content and content creators on influential social media platforms. These selected contents, processed through heuristic cues, serve as vital sources of information, guiding destination selection and reinforcing the tendency to imitate others' actions [107]. The intricate interplay of induced information and influences leads to the emulation of other travelers' behavior [69].

Additionally, the dynamic dual-process framework further emphasizes the complexity of decision-making among American millennials in a digitally connected society. Particularly in contemporary destination selection scenarios, this multifaceted process involves the simultaneous evaluation of multiple factors, all of which influence the final decision.

### 6.2 Theoretical implications

The validated multidimensional model provides a robust framework for exploring and understanding the bandwagon effect across diverse contexts, supporting the suggestions of Boto-García and Baños-Pino [24] and Nadroo, Lim [76] regarding multiple mechanisms underlying the bandwagon effect in both tourism and consumer behavior. This approach contrasts with the single-dimensional models often used in consumer behavior studies, like those by Anantharaman, Prashar [70] and Eastman and Iyer [71]. The model offers insights into how limited cognitive capacity and expert influences psychologically formulate and influence behaviors across various social scenarios. The established utility of the complex model construct of the bandwagon effect suggests its potential applicability in future studies, not only within the domain of tourism but also in other related fields of social and behavioral sciences concerning human behavior where the influence of pertinent others exerts relevance.

The structural model emphasizes the applications of the bandwagon effect and underscores the crucial roles of attitude and subjective norm as core determinants in the model framework, both in direct and mediating roles. It is reasonable to infer that decision-making involves a dynamic internal process, wherein a sequence of preferences and beliefs evolves through different stages before culminating in behavioral intention [111].

Limited research in tourism has attempted to clarify dual-process mechanisms within a unified model that integrates cognitive bias factors from behavioral economics and the rational approach theory of planned behavior. Building on the perspectives of Chaiken and Ledgerwood [32] and McCabe, Li [29], this study proposes that the travel-related decision-making process can be explained by a co-existing dual-system, incorporating both systematic processing and simplification. This dual process theory suggests that both a logically rational approach and a preference-based bounded rationality approach are simultaneously employed in information and decision processes [31,123,124].

The study emphasizes how intuitive, low-involvement principle and deliberative, high involvement rationally processes are concurrently engaged and contributes to an alternative decision-making framework [33,35]. This understanding can further facilitate an improved framework for investigating travel-related decision-making models [124].

### 6.3 Practical implications

Acknowledging the significant impact of social media and influencer marketing on millennials' travel behaviors, destination marketers have increasingly leveraged these platforms to promote destinations. This approach has the potential to influence travelers' behavior, particularly in how they process information for destination selection [77,84]. Millennials, recognized as a crucial demographic with considerable potential for expanding Thailand tourism market, demonstrate a distinct decision-making process when selecting holiday destinations.

A reexamination of the proposed model in the aftermath of a global crisis holds significance for reassessing the determinants shaping millennials' behavior. This study underscores the enduring influence of social media peers and reference groups, even as cohorts mature into professionals with established careers and families. The results also highlight gender and education disparities, indicating that men and individuals with higher education are more susceptible to the bandwagon effect within systematic processes.

In crafting destination communication strategies, destination management should prioritize and highlight the applications of the bandwagon effect, which accentuates the significance of pertinent contents and their creators in shaping millennials' travel consumptions [86]. Generic destination promotion content or creators may not yield the desired results. Utilizing current, well-known figures who resonate with the target audience to convey carefully crafted, audience-specific messages can significantly increase the appeal and choice of the destination. Tailored content from relevant creators on suitable platforms could significantly prompt and impact millennials social comparison [96] and perceived social return [21], as travel experiences are impartially valued.

Particularly with the algorithm of social media screening and selecting what travelers see based on set relevant factors according to their need for aspiration [96], belonging and identification [91] as members of social groups. The salience of considering relevance is paramount, especially in discerning group affiliation and identifying influential figures within those groups. Destination management must actively engage in acquiring, utilizing, supplying, and sharing tourism-related content [92], with a series of content designed to amplify and maintain the continuity of the bandwagon effect [70].

Liu and Chong [14] study suggests to combine the use of both official and user generated content, however, as noted by Bonilla-Quijada, Tugores-Ques [127] and Liu and Chong [14], advertising content from official accounts is less effective than content generated by popular creators, so the marketing communication strategics to employ highly engaged and relevant content creators for a targeted group should be prioritized.

Moreover, persuasive content promoting Thailand as a destination should prioritize positive testimonials on expected outcomes [17] and emphasize endorsements and feedback from influential figures within travelers' expanding social circles, particularly among male audiences. Given that social influence on travel intentions is mediated by attitudes and subjective norms, showcasing authentic, positive experiences can effectively encourage future interest in visiting, especially among male millennial target audiences.

Additionally, prioritizing the assessment of a destination's fitness, which involves evaluating how effectively content and creators capture and align with destination peculiarity [77,84], is essential. This can be achieved by gaining insights into visitors' perceptions and expectations of the destination. For instance, research on Thailand indicates that the majority of American millennials perceive destinations as entertaining and affordable, prioritizing these aspects over others. Ensuring a strong alignment between content and destination is crucial, as it has the potential to maximize impact.

Moreover, as emphasized by Beall, Boley [80], Han, Wang [107], and Sun and Xing [102], leveraging persuasive influence can further guide targeted audiences toward desirable outcomes. The bandwagon effect and subjective norm can be effectively employed to promote desirable concepts such as sustainability, ecotourism, or cultural tourism.

### 6.4 Limitations and future studies

Online surveys and convenience sampling offer significant convenience and efficiency, especially for digitally savvy Millennials. However, the absence of direct engagement offered in qualitative methods can potentially impact response consistency and result in a limited understanding of the questions. Additionally, selection bias may arise from non-random sampling.

Acknowledging that only the bandwagon effect served as a heuristic and cognitive bias factor in this study, it is recognized that the model may not comprehensively nor completely illuminate the intricate dynamics of the underlying decision-making process. Nonetheless, examining this selected factor proves beneficial in enhancing our comprehension of its intricacies and the associated underlying processes.

Moreover, it serves as an exemplar of the incorporated and dual process model. Future research endeavors may explore additional heuristic and cognitive bias factors, thereby improving the model's explanatory and predictive capacity. Furthermore, conducting a longitudinal study of American Millennials is encouraged to analyze the effects over time. Additionally, this framework can be applied to other nationalities and destinations.

## CRediT authorship contribution statement

**Roannayutt Oan-Oon:** Writing – review & editing, Writing – original draft, Methodology, Investigation, Formal analysis, Conceptualization. **Therdchai Choibamroong:** Supervision.

## Data available statement

Due to the conditions set by the ethics approval governing this research, the data collected is not available for public access or distribution. Access to the data is restricted solely to the principal investigator.

## Declaration of competing interest

The authors declare that they have no known competing financial interests or personal relationships that could have appeared to influence the work reported in this paper.
