# Supplementary material for: Multidimensional bandwagon effect and dual-process decision making: An integrated model of the theory of planned behavior from the perspective of American millennials’ travel intention
Source: Heliyon. 2024 Dec 21;11(1):e41375. doi: 10.1016/j.heliyon.2024.e41375 (PMC11732704; doi:10.1016/j.heliyon.2024.e41375)
Supplement: Multimedia component 1 [file mmc1.pdf]

Research questionnaire

Section 1.1 Screening questions

1)Are you planning international travel in the next 2 years?	Yes
	No
2)Have you been to Thailand?	Yes
	No
3)How often you have seen social media content about traveling to Thailand?	Often
	Sometimes
	Rarely

Section 1.2 Generation information

1)Age group	Less than 28
	28- 34
	35-43
	over 43
2)How do you describe your gender?	Man
	Woman
	Transgender
	Prefer not to answer
3) Marital status	Single
	With partner with childern
	With partner no childern
	Separated / Divorced / Widowed
4)Education level	High school
	College degree
	Advanced degree
5)Annual Household Income	Less Than USD 50,000
	Between USD 50,000 -150,000
	Between 150,000-350,000
	More Than 350,000
6)Occupation	Unemployed
	Entrepreneur
	Full time employee
	Part time employee
	Retired

Bandwagon cue

	Very unlikely	Unlikely	Somewhat Unlikely	Neutral	Somewhat Likely	Likely	Very likely
1) I'm likely to be interested in social media contents about traveling to Thailand because the popularity of the content as represents by level of content engagement such as number of likes, comments & shares.							
2) I'm likely to interested in social media contents about traveling to Thailand because the popularity of the content creator as represented by number of subscribers & followers.							
3) I'm likely to interested in social media contents about traveling to Thailand because the popularity of the topic as current and emerging trend							
4) I'm likely to interested in social media contents about traveling to Thailand because the popularity of the social media platform							

Normative influence

	Strongly disagree	Disagree	Somewhat disagree	Neither agree or disagree	Somewhat agree	Agree	Strongly agree
1) I'm interested to travel to Thailand after seeing others' experiences of visiting on social media because following their actions is likely to result in my inclusion within social group.							
2) I'm interested to travel to Thailand after seeing others' experiences of visiting on social media because following their actions is likely to result in my visibility within social group							
3) I'm interested to travel to Thailand after seeing others' experiences of visiting on social media because I feel pressured to follow others							

Informational influence

	Strongly disagree	Disagree	Somewhat disagree	Neither agree or disagree	Somewhat agree	Agree	Strongly agree
1) Social media contents are important source of information about traveling to Thailand because I believe that information to be credible							
2) Social media contents are important source of information about traveling to Thailand because people who have visited have more knowledge than I do							
3) Social media contents are important source of information about traveling to Thailand because I think information helps me deciding between choices.							
4) Social media contents are important source of information about traveling to Thailand because I value opinions and recommendations of others.							

ATTITUDE

	Not at all Enjoyable						Very Enjoyable
1) All things consider, I. personally think visiting Thailand would be	1	2	3	4	5	6	7

	Not at all Entertaining						Very Entertaining
2) All things consider, I personally think visiting Thailand would be	1	2	3	4	5	6	7

	Not at all Adventurous						Very Adventurous
3) All things consider, I personally think visiting Thailand would be	1	2	3	4	5	6	7

	Not at all Value for money						Very Value for money
4) For me, a trip to Thailand would be	1	2	3	4	5	6	7

	Not at all Authentic						Very Authentic
5) For me, a trip to Thailand would be	1	2	3	4	5	6	7

	Not at all Serene						Very Serene
6) For me, a trip to Thailand will be	1	2	3	4	5	6	7

SUBJECTIVE NORM

	Strongly disagree	Disagree	Somewhat disagree	Neither agree or disagree	Somewhat agree	Agree	Strongly agree
1) People who are relevant to me think I should visit Thailand.							
2) People who are relevant to me would approve of me visiting Thailand.							
3) People who are relevant to me would visit Thailand themselves							

INTENTION TO VISIT THAILAND

	Strongly disagree	Disagree	Somewhat disagree	Neither agree or disagree	Somewhat agree	Agree	Strongly agree
1) Thailand would be among my first choice of destinations.							
2) I will visit Thailand in the future.							
3) I will save time and money for the purpose of visiting Thailand.							
